# High‐throughput mechano‐cytometry as a method to detect apoptosis, necroptosis, and ferroptosis

**DOI:** 10.1111/cpr.13445

**Published:** 2023-03-29

**Authors:** Louis Van der Meeren, Joost Verduijn, Dmitri V. Krysko, André G. Skirtach

**Affiliations:** ^1^ Nano‐BioTechnology Laboratory, Department of Biotechnology, Faculty of Bioscience Engineering Ghent University Ghent Belgium; ^2^ Cancer Research Institute Ghent Ghent Belgium; ^3^ Cell Death Investigation and Therapy Laboratory, Department of Human Structure and Repair, Faculty of Medicine and Health Sciences Ghent University Ghent Belgium

## Abstract

In recent years, the importance of the investigation of regulated cell death (RCD) has significantly increased and different methods are proposed for the detection of RCD including biochemical as well as fluorescence assays. Researchers have shown that early stages of cell death could be detected by using AFM. Although AFM offers a high single‐cell resolution and sensitivity, the throughput (<100 cells/h) limits a broad range of biomedical applications of this technique. Here, a microfluidics‐based mechanobiology technique, named shear flow deformability cytometry (sDC), is used to investigate and distinguish dying cells from viable cells purely based on their mechanical properties. Three different RCD modalities (i.e., apoptosis, necroptosis, and ferroptosis) are induced in L929sAhFas cells and analysed using sDC. Using machine learning on the extracted parameters, it was possible to predict the dead or viable state with 92% validation accuracy. A significant decrease in elasticity can be noticed for each of these RCD modalities by analysing the deformation of the dying cells. Analysis of morphological characteristics such as cell size and membrane irregularities also indicated significant differences in the RCD induced cells versus control cells. These results highlight the importance of mechanical properties during RCD and the significance of label‐free techniques, such as sDC, which can be used to detect regulated cell death and can be further linked with sorting of live and dead cells.

## Graphical abstract

In this work, we investigate the viability to use a microfluidics‐based approach, shear flow deformability cytometry (sDC), to measure mechanical properties of cells undergoing regulated cell death. Three different regulated cell death modalities were observed. Using machine learning prediction on the parameters extracted from sDC, it was possible to predict with 92% accuracy the live/dead state of cells. The main difference between live and dead cells showed to be the deformation of the cells which is strongly linked to the mechanical state of cells.

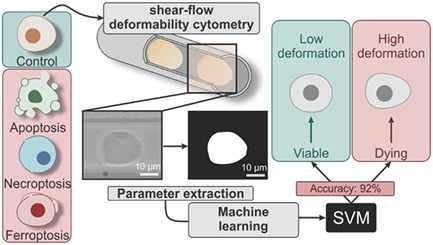

## 1 INTRODUCTION

Regulated cell death (RCD) is defined by the Nomenclature Committee on Cell Death (NCCD) as ‘any form of cell death that results from the activation of one or more signal transduction modules, and hence can be pharmacologically or genetically modulated’.^1^ The first identified, and consequentially most thoroughly studied, form of RCD is apoptosis which is introduced in 1972 by Kerr et al.^2^ However, in recent years an increasing number of RCD modalities have been discovered among which, but not limited to necroptosis (a RCD which mainly is regulated by the kinase proteins, RIPK1 and RIPK3, and dependant on their substrate MLKL for the execution) and ferroptosis (an iron‐dependant RCD that results from the lethal accumulation of peroxidised lipids).^3^ Most of these RCD modalities have shown to play an important role in several diseases,^4^, ^5^, ^6^ which has led to an enormous increase of interest in the cell death field. One of the best‐known examples is the evasion of apoptosis in tumour development, which has been defined as one of the hallmarks of cancer,^7^ other important examples are, but not limited to, the role of different RCD modalities in heart diseases,^8^ neurological disorders^9^ and the role of different RCD modalities in the development of immune responses.^10^, ^11^

When researching the potential of drugs or other compounds to induce cell death, a common strategy is to use fluorescent dyes which mark the occurrence of an essential step in the cell death process to indicate cellular membrane permeabilization (e.g., propidium iodide or Sytox green). However, these markers also entail some limitations, among which, prolonged excitation of fluorescent dyes which can lead to phototoxicity,^12^ markers often only indicate later stages of cell death and fluorescent labels, in some cases, do not allow for discrimination in between different modalities.^13^ To overcome these limitations label‐free microscopy techniques have gained increasing interest.

**FIGURE 1:**
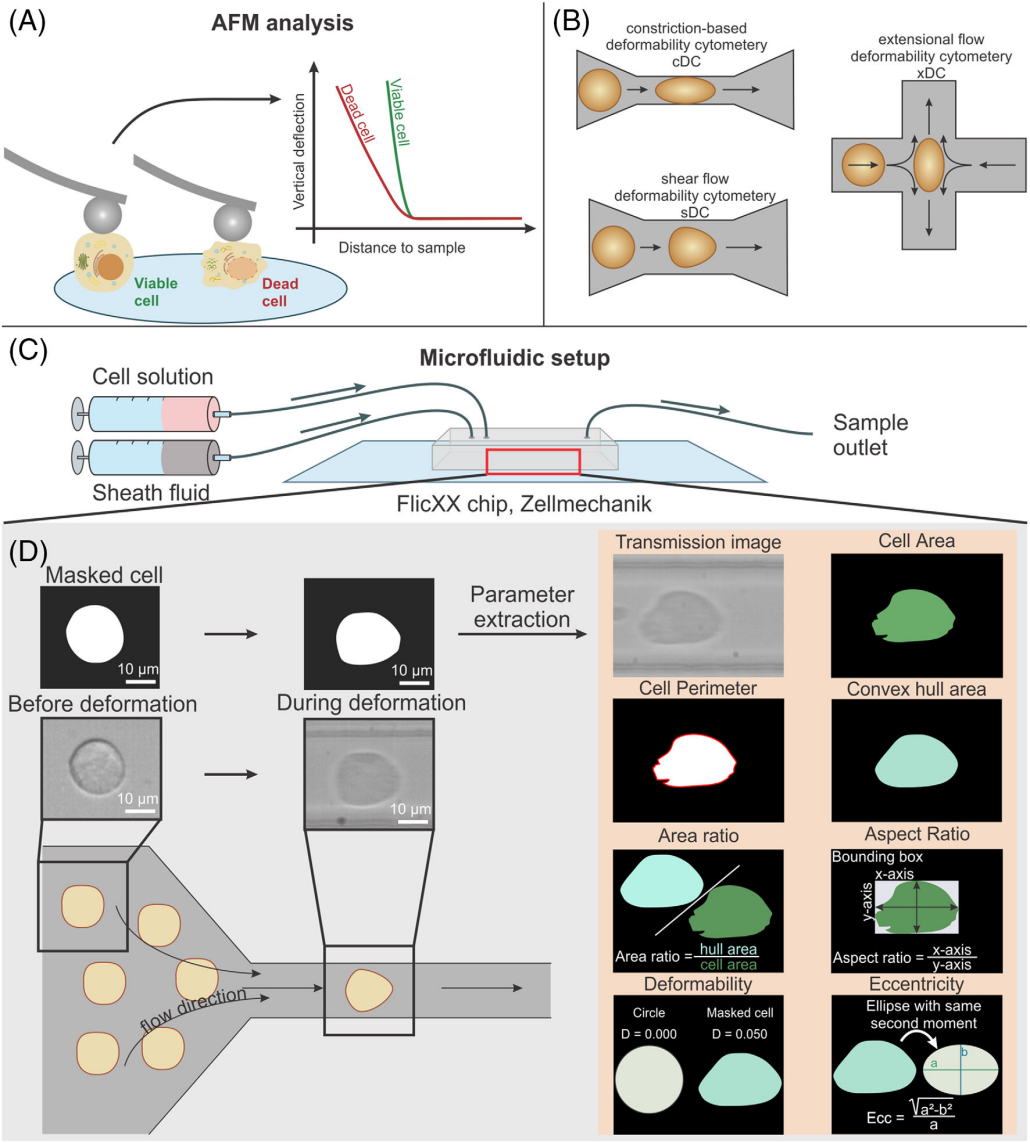
Schematic representation of the experimental setup of the sDC analysis. (A) A schematic representation of AFM measurements of viable cells and cells undergoing RCD measuring drop of elasticity resulting in larger indentations, which translates to a lower elasticity as expressed in Young's modulus. (B) A schematic representation of different microfluidic‐based high throughput mechano‐cytometers. Constriction‐based deformability cytometry (cDC): channel is narrower than the cell diameter, the time needed to cross the channel is measured. Shear flow deformability cytometry (sDC): the channel is slightly bigger than the cell diameter and deformation caused by channel shear force is measured. Extensional flow deformability cytometry (xDC): deformability perpendicular to the direction of flow is analysed. (C) Macroscopic view of the sDC experimental setup. Two separate syringes, one filled with cells suspended in carrier fluid and the other with pure carrier fluid are mounted onto syringe pumps and connected to separate inlets of the DChip. On the other side of the chip, tubing is connected to the chip outlet allowing to reuse or discard of the sample. (D) Zoomed‐in view of the constricting channel of the sDC chip, both sheath solution and cell solution join each other at the constriction channel. Due to the speed of the solutions and the shear forces exerted on the cells through the viscous fluid and the narrow channel walls (30 μm), cells are deformed. The cells flowing through this channel are captured using a high‐speed CCD camera. From these images a multitude of different parameters can be extracted which of which the calculations are shown on the right. Parameters such as area, perimeter and area ratio offer information on the morphological state of the cells and aspect ratio, deformability and eccentricity give insight into the mechanical state of the cell. A more detailed explanation of the parameter extraction is provided in Section 2.

In the past decades, a range of different strategies to analyse regulated cell death in a label‐free manner have been emerging among which are Raman spectroscopy,^14^ digital holography microscopy (DHM)^15^ and mechanobiology analysis.^16^ A range of biological states and processes have previously been associated with changes in mechanical properties.^17^ Atomic force microscopy (AFM), a nanoindenter tool initially developed for material science applications, can be used to analyse the mechanical properties of soft samples. An example of this is found in a paper analysing the mechanical properties of polymeric capsules,^18^ this data could then be used to derive the forces that cells exert on such capsules.^19^ Recent developments in AFM techniques have greatly increased the use of this technique in imaging and mechanical measurements of biological samples.^20^, ^21^, ^22^, ^23^ It has been reported that a distinction between different modes of RCD could be made by comparing dynamics in elasticity and microrheology, using AFM (Figure 1A).^16^ However, the limitation inherently connected to the AFM technique is a low throughput. To improve this, techniques are being developed that allow to analyse mechanical properties of cells at a higher throughput. Often a microfluidics approach is used which limits free movement of the cell in order to analyse deformation.^24^ This technique is the mechanical simile to flowcytometry, a technique well engrained in cell death analysis.^25^ Some examples of this approach include constriction‐based deformability cytometry (cDC),^26^ shear flow deformability cytometry (sDC)^27^ and extensional flow deformability cytometry (xDC)^28^ (schematic representations of these techniques are shown in Figure 1B). Although all these techniques show promising results and allow throughputs up to 200 cells per second, they have not been applied to identify induction of RCD.

Even though the implementations of these high throughput methods are quite recent, there is a surge studies that are looking into possible applications of these methods. A first possible type of application is the analysis of the effect of drugs on mechanobiology. Examples of this are shown in research on the effect of cytoskeletal drugs in a human leukaemia cell line,^29^ the effect of a V‐ATPase inhibitor on the mechanical properties of cancer cells with the outlook of developing new treatment strategies^30^ and treatment of Chorea‐acanthocytosis with Dasatinib or Lithium.^31^ Furthermore, deformability cytometry can be used to characterize changes of cellular states such as maturation of dendritic cells^32^ and differentiation of pluripotent stem cells^33^ or changes in mechanical properties due to certain pathologies as was done for *Plasmodium falciparum* infection.^34^ Besides its use for fundamental research purposes, several papers have also been already exploring more applied uses for this technique, examples are the quality assessment of cellular blood products^35^ and the differentiation between healthy and tumorous tissue in biopsy samples using a machine learning based analysis.^36^

It was reported that sDC and cDC work at similar low strain rated while xDC applies higher strain rates, therefore cDC or sDC were posed as most suited to measure changes in the actin cytoskeleton,^24^ since at higher strain rates the actin cytoskeleton can fluidize. Considering the knowledge gained from previous research on the changes in mechanical properties of cells due to breakdown dynamics these two techniques are most promising.^16^ Of note that during induction of regulated cell death, cells detach from the substrate and tend to agglomerate, which would lead to an increased risk of channel clogging in case of cDC compared to sDC. Therefore, here, a sDC setup is used to analyse the mechanical changes during RCD. For sDC two syringe pumps, one flowing sheath fluid while another has cells suspended in sheath fluid, are connected to a specialized chip that contains a narrow channel on where cells are focused through (Figure 1C). At the end of that channel, a transmission image is acquired. By performing automated object detection on these images, the cell can be distinguished from the background and a mask can be created. This mask is then used to extract a multitude of parameters from the cell (Figure 1D). It is hypothesized that by inducing RCD in cancer cells, a significant drop in cellular elasticity will occur, which will be measurable by analysing the change in deformability of the cells flowing through the narrow channel allowing to distinguish these cells from control viable cells. Furthermore, cells undergoing RCD show characteristic changes in morphology which can further help distinguish the dying cells from the control cells.

## 2 MATERIALS AND METHODS

### 2.1 Cell culture and cell death induction

Murine fibrosarcoma L929sAhFas cells were cultured in Dulbecco's Modified Eagle Medium (LONZA, 12‐604F) supplemented with 10% FBS (FisherScientific, 11591821), 1% Penicillin/streptomycin (LONZA, DE17‐602E). Apoptosis is induced by adding 2.5 μM of Staurosporin (Sigma‐Aldrich, S5921). Necroptosis was induced by adding tumour necrosis factor alpha, mTNFα (recombinant protein from VIB Protein Service Facility, 250 ng/mL). Ferroptosis was induced in this cell line by adding ML162 (SanBio, 20455‐5, 10 μM). MCA205 cells were cultured in RPMI 1640 (LONZA, BE12‐702F) supplemented with 10% FBS (FisherScientific, 11591821), 1% Penicillin/streptomycin (LONZA, DE17‐602E).

### 2.2 Induction of RCD and time kinetics analysis

With the aim of investigating the kinetics of the RCD modalities, an overtime induction experiment was performed. Cells were seeded in a 96‐well plate at a concentration of 10,000 cells per well 24 h prior to the experiment. Cell death induction was performed using the mentioned above concentrations. These cells were imaged every hour using fluorescence microscopy (Ti‐e, Nikon). Two fluorescent labels were added to the cells to allow analysis of the cell state. Hoechst33342 (1 μM, Thermofisher, H3570) was added to visualize the nucleus of all cells, Propidium Iodide (1 μM, Thermofisher, P1304MP) is added allowing to visualize the nuclei of dead cells (where membrane permeabilization has occurred). The blue channel (Hoechst33342) and red channel images (Propidium iodide) can be overlayed to calculate the percentage of dead cells. All image processing for these experiments was performed using Fiji and custom scripts (V. 1.53s).

### 2.3 Analysis of mechanical properties by AFM

To analyse the mechanical properties of the cells, AFM was used. The cells were trypsinized right before the experiment and seeded in a confocal imaging dish (VWR) to a density of 500,000 cells/mL. To prevent any changes in mechanical properties due to formation of stress fibres (involved in cell attachment), the mechanical properties were measured immediately after adding the cells to the dish (while still in suspension). The AFM instrument is a Nanowizard 4 BioAFM (JPK bioAFM, Bruker). The measurements were performed in a liquid environment using an in‐house‐made colloidal probe with the radius of 5 μm. To extract the Young's modulus, the obtained force curves were processed in the JPK DP software using an adjusted Hertz model for spherical probes.

### 2.4 Deformability cytometry

The cells were prepared for the sDC as follows. First, the cells are trypsinized. Subsequently, the cell suspension is strained through a mesh of 40 μm to exclude large cell agglomerations. Cells are then, centrifuged at 200*g* for 5 min and subsequently resuspended them in a specialized buffer fluid with a higher viscosity for the sDC (CellCarrier, Zellmechanik GmbH, Dresden, DE). A deformability cytometry setup was made according to previously published data by Otto et al.^37^ Two separate syringe pumps (Harvard apparatus PhD ultra 70‐3006 and model 22) were used to drive both sample and sheath fluid flow. The sample and sheath fluid were flowed at a speed of 0.03375 and 0.10125 μL/s, respectively. These syringes were connected using tubing to a specialized microfluidic chip (polydimethylsiloxane on borosilicate glass) for sDC with a channel of 30 μM (Flic30, Zellmechanik GmbH, Dresden, DE). The DC setup was mounted onto a Nikon TE2000 setup. Images were acquired using a highspeed CMOS camera (QVIT, AOS technologies) at speeds higher than 3000 frames per second Post‐processing of the captured images was performed using a personal script in MATLAB (v. 2022; Supporting Information S1). During the post‐processing, a size filter is applied to objects with an area between 78 μm^2^ which is the reported upper size limit of apoptotic bodies^38^ and 284 μm^2^ which is the equivalent area of a circle with a diameter of 40 μm^2^, the mesh size used in the previous filtration.

The following six parameters are isolated for cells in these experiments: area, perimeter, area ratio, deformability, eccentricity, and aspect ratio (Figure 1D, Table S1). Area, perimeter, and area ratio are related to the morphological state of the cells. The area ratio offers information on the roughness of a cell. Higher values indicate a bigger difference between the actual cell area and the convex hull around the cell, to prevent that doublets of cells or objects that are too irregular are included in the dataset a threshold value is set at 1.2, these values are based on previously reported data.^39^ Detected objects with an area ratio higher than this value are excluded from the further analysis. Deformability, eccentricity, and aspect ratio, on the other hand, offer information on the mechanical state of the cells. Deformability, defined as 1‐circularity (a measure of how much an object deviates from a circle, with a circle being the extreme case: 0) is an indicator for cellular elasticity,^40^ higher deformability indicates a softer cell and vice versa. Eccentricity is another measure for the elongation of an object, this value is calculated from an ellipse with the same second moments as the cell mask. Eccentricity offers information on the elongation and has two degenerate cases: 0 for a circle and 1 for a line. The aspect ratio is a less accurate measurement of elongation which takes the ratio of the *x*‐axis over the *y*‐axis of the bounding box around the detected cell (a more detailed calculation of these parameters is shown in Table S1).

The aforementioned parameters are not completely independent of each other. An important consideration that needs to be made is that objects with a bigger area, but the same elasticity will show a larger deformability when flown through the same channel diameter. Another parameter that should be interpreted carefully is area ratio. Normally control cells are smooth which results in an area ratio close to 1. Irregularities on cells, such as blebs, will increase this value. In relation to that, deformability is artificially increased by these irregularities (since it is calculated from both perimeter and area). Eccentricity, on the other hand, is less dependent on these irregularities. Taking this into account, it has previously been suggested that at the threshold of an area ratio higher than 1.05, alternative parameters describing elongation should be preferred over deformability for the interpretation of mechanical properties of the cell,^41^ in this work, eccentricity is preferred when this threshold has been crossed.

### 2.5 Live/dead cell pre‐filtering using support vector machine classification model

To pre‐filter the data before data analysis into control and dead/dying cells a classification support vector machine (SVM) model was built on a subset of 400 datapoints (200 control and 200 dead/dying cells) for L929sAhFas cells and 200 datapoints (100 control and 100 dead/dying) for MCA205 cells. These models are built using the Classification learner application available in MATLAB (version 2022a 9.12.0.1884302). Using this application an optimized neural network was built.

### 2.6 Data processing and graphs

Graphs are produced using Origin Pro (v. 2021). All statistical analyses were performed using R studio (v. 2022.02.3). Depending on the normality of the data or size of the sample sets appropriate statistical tests were applied.

## 3 RESULTS AND DISCUSSION

### 3.1 Changes in mechanical properties of suspension cells after induction of RCD using AFM

**FIGURE 2:**
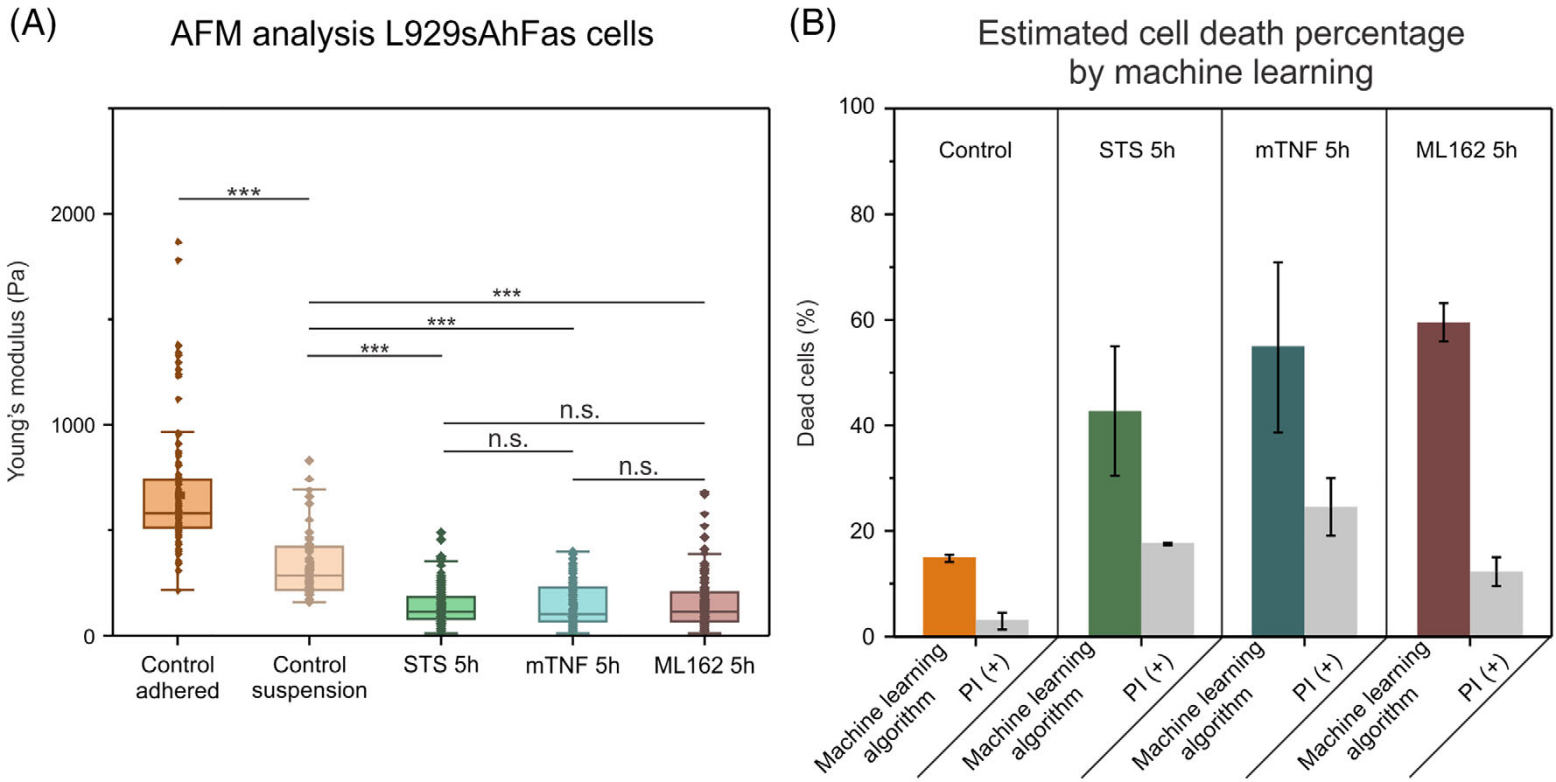
Mechanical characterization and machine learning prediction of RCD. (A) Mechanical properties of L929sAhFas cells undergoing different RCD modalities. Measurements are made using an AFM equipped with a colloidal probe. For all three RCD modalities, a significant decrease in elasticity can be reported (***p < 0.001, n.s.: non‐significant). (B) Bar graph representing the percentages of the amount of dying/dead cells in the sDC experiments as (dark bars) estimated by the classification of a SVM model trained on L929sAhFas cells (light bars) by fluorescence microscopy indicated by PI(+).

The difference in elasticity between viable and dead cells was assessed using a well‐established method namely AFM. In this work, a specific adherent cell line namely murine fibrosarcoma L929sAhFas is used which allows to induce three different RCD modalities (i.e., apoptosis, necroptosis and ferroptosis) in a similar timeframe by adding different inducers.^15^, ^16^, ^42^ Since the sDC setup requires cells to be in suspension, the L929sAhFas cells were brought into a suspension state by trypsinization after which a significant drop is observed in the Young's modulus of the control (viable) cells (a parameter commonly used to represent elasticity) from 669 Pa (±293.06 Pa) to 300 Pa (±156.10 Pa; Figure 2A). Next, it is analysed if induction of RCD still resulted in a significant change of elasticity after cells are brought into suspension. For these experiments, a particular type of RCD is induced in the L929sAhFas cells, and after 5 h, the cells are detached, and their mechanical properties are measured. The Youngs modulus of more than 20 cells per group is analysed (Figure 2A). We have found that for all induced RCD modalities, a significant drop in elasticity is observed when compared to control viable suspension cells (*p* < 0.001, Kruskal–Wallis test). However, no significant difference in elasticity between the RCD modalities have been observed.

The AFM analysis showed that the Young's modulus of adherent control L929sAhFas is approximately 700 Pa, whereas the L929sAhFas control cells in the suspension have a significantly lower average value of the Young's modulus (336.7 Pa). It has been reported previously that cellular elasticity decreases due to cellular detachment.^43^ The focal adhesion points, and more specifically the stress fibres that originate in these points, are disturbed during cellular detachment (e.g., by enzymatic detachment). Given that detaching cells from the surface already leads to a decrease in elasticity, here, still, a significant decrease can be observed when RCD is induced in the cells. On average, the Young's modulus of the control viable cells is halved (from *±*300 Pa to *±*150 Pa upon RCD induction). Importantly, this difference in elasticity is essential for the next steps in the analysis of this work. Since it has been established that the stress fibres and the amount of them can strongly affect the cellular elasticity,^44^ it is to be expected that detached cells will still show a decreased elasticity after induction of RCD. In between the different cell death modalities, no significant differences in Young's modulus are reported. This is to be expected since the previously reported differences between these modalities occur at earlier stages during the cell death process than are investigated in this work.^16^

### 3.2 Live/dead cell filtering using support vector model

Due to an inherent presence of heterogeneity during RCD induction, not all cells will enter the cell death process at the same time. These variations can depend on many parameters, among which, the cellular micro‐environment, cell density or the stage of cells in the cell‐cycle.^45^, ^46^ To get an idea how this is present in the L929sAhFas cell line, a fluorescence microscopy kinetics experiment was performed (Figure S1). For this experiment, a fluorescent label is used that robustly indicates end‐stage cell death for apoptosis, necroptosis and ferroptosis, which is propidium iodide. High percentages of end‐stage cell death are only observed after 24 h (95%, 88%, and 98% for apoptosis, necroptosis, and ferroptosis, respectively) which are not a sudden switch but rather a gradual increase. The question posed here is if sDC can be used to detect the onset of cell death by analysing the mechanical properties. We have found a significant decrease in elasticity after 5 h for apoptosis, necroptosis, and ferroptosis. Importantly at this time point, only a low percentage of cells are PI positive (17% apoptosis, 24% necroptosis, and 12% ferroptosis; Figure 2B), while their Young's modulus has decreased significantly (Figure 2A). These data suggest that the sDC analysis can be also performed on dying cancer cells 5 h after cell death induction.

**FIGURE 3:**
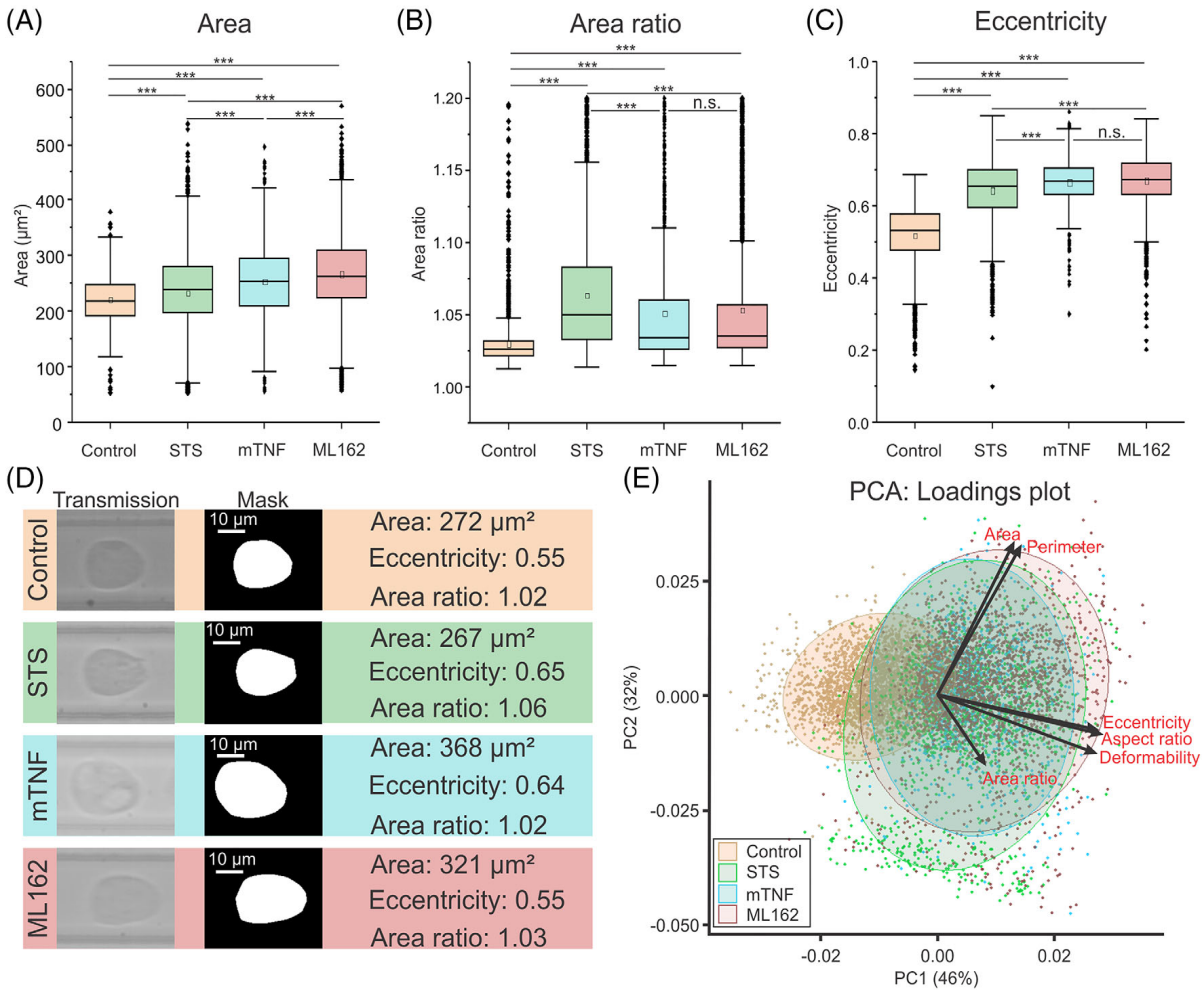
sDC analysis of L929sAhFas cells undergoing apoptosis (STS), necroptosis (mTNF) and ferroptosis (ML162). (A) Boxplot graph comparing the area (μm^2^) of cells after 5 h of induction of apoptosis (2.5 μM staurosporin), necroptosis (250 ng/mL mTNF), and ferroptosis (10 μM ML162). (B) Boxplot graph comparing area ratio of cells undergoing apoptosis, necroptosis, and ferroptosis. (C) Boxplot graph comparing the eccentricity of cells at different time points undergoing apoptosis, necroptosis, and ferroptosis. (Statistical significance indications are compared to control cells, ***p < 0.001, n.s., not significant, *p* > 0.05. Significance calculated by pairwise *t*‐tests using Bonferroni correction). (D) Exemplary images of cell and their corresponding masks from each time point analysed during apoptosis, necroptosis and ferroptosis induction, the values indicated next to the cells are directly derived from these masks. (E) PCA plot: the dots indicate the individual cells while the transparent ellipse represents the 90% confidence interval of the data, dots are plotted on the *x*‐axis following the first PC and on the *y*‐axis following the second PC, the arrows indicate the eigenvectors of the parameters.

Since not all cells that are measured at a certain time point will be dead or have already entered the cell death process, it is important to make a distinction between cells that are still considered viable and cells that are dying or dead. To achieve this prediction a classification of sDC data has been performed. With the aim of building a model based on a small subset of labelled data which is subsequently used to predict the viability state of the other cells that were collected (this is similar to a previously published study using DHM^15^). Initially, 150 cells were selected from a control experiment (i.e., cells where no cell death inducer was added) were selected knowing the average size of control L929sAhFas cells (a cell area approximating 230 μm^2^, as determined from fluorescence imaging, Figure S2). Combined with the knowledge from other studies in which sDC^37^ has been used, control cells show a smooth contour with few irregularities, an example of such a viable cell is shown in Figure 3D. Next, taking this into account, 50 cells were selected of each condition where cell death was induced. Those cells were selected that clearly differed visually from the average control viable cell that could be observed in the control cell experiments. With this subset, a SVM could be built on the extracted parameters of these cells, resulting in a model with a validation accuracy of approximately 92%, using the classification learner app in MATLAB (using a 5‐fold cross‐validation). This model was subsequently exported into a classifier function (script of this function is shown in Supporting Information S2). Applying this model to the remainder of the data showed an average of 14% dead cells in control condition, while for the other conditions, the estimated dead cell percentages are: 42% (apoptosis), 54% (necroptosis), and 60% (ferroptosis; Figure 2B).

These results indicate that after 5 h of adding the inducers approximately 52% of cells are estimated as dead or dying. When comparing this to the results obtained from the fluorescent cell death kinetics experiments where an average of approximately 18% dead cells can be labelled as dead based on the analysis of the plasma membrane integrity by PI (18% apoptosis, 25% necroptosis, and 12% ferroptosis). This data suggests that sDC allows for potential detection of early‐stage cell death based on data related to the mechanical properties. This is in line with previous research on the mechanical properties of cells undergoing cell death.^47^ For control cells, an average estimated 14% of cells were classified as dead. This value is higher compared to that obtained from the fluorescence microscopy experiments (3%). However, these are some factors that potentially affect this number. First, L929sAhFas cells are inherently adhesive cells, so in order to detach them from the substrate these need to be treated with trypsin. Previous research on the damaging effect of different cell harvesting methods reported an average cell death of 10% after trypsin treatment, which is in line with the results presented here.^48^

### 3.3 Comparison of the effects caused by apoptosis, necroptosis, and ferroptosis in L929sAhFas cells using deformability cytometry

The data of the extracted parameters, after filtering the data for all the RCD modalities, is presented in Table 1 (area, perimeter, deformability, area ratio, eccentricity, aspect ratio; a detailed explanation of these parameters see in Section 2 while statistical information is shown in Table S2). To give an indication of how a representative cell looks during the different RCD modalities, three cells are represented in Figure 3D with the corresponding values of area, area ratio and eccentricity.

**TABLE 1:** A summary of area, perimeter, deformability, area ratio, eccentricity, and aspect ratio per condition which were analysed in the L929sAhFas cells (± indicates standard deviation).

	Control	Apoptosis (STS)	Necroptosis (mTNF)	Ferroptosis (ML162)
Cell area	221 (±40.6)	233 (±78.4)	252 (±67.2)	281 (±73.8)
Cell perimeter	52 (±5.0)	54 (±10)	57 (±7.9)	60 (±8.6)
Area ratio	1.030 (±0.017)	1.064 (±0.041)	1.051 (±0.040)	1.062 (±0.045)
Deformability	0.013 (±0.004)	0.035 (±0.011)	0.033 (±0.010)	0.035 (±0.011)
Eccentricity	0.52 (±0.081)	0.64 (±0.085)	0.67 (±0.063)	0.64 (±0.074)
Aspect ratio	1.19 (±0.07)	1.37 (±0.14)	1.37 (±0.10)	1.35 (±0.10)

Looking first at the area (Figure 3A) and perimeter, it can be observed that for all RCD modalities a significant increase is observed compared to the control (*p* < 0.001; Table 2). Further intercomparing the different RCD modalities between each other also shows significant differences in the area and perimeter. The smallest increase of area and perimeter is noticed for apoptotic cells (233 μm^2^) which are significantly smaller than necroptotic cells (252 μm^2^) which on their turn are significantly smaller than ferroptotic cells (281 μm^2^; *p* < 0.001, Table S2). While no clear reports exist of cells swelling during apoptosis, it is possible that this small area increase is caused by the presence of membrane blebs on the cellular surface, most likely caused by membrane destabilization.^49^ Another characteristic that can be noticed about the cell area for apoptotic cells is that a small subpopulation exists with a much lower area than those at all other conditions (Figure 3E). A potential explanation for this sub‐population could be that these are apoptotic bodies originating from the apoptotic cells or debris released from apoptotic cells. During necroptosis, the pore‐forming molecule, mixed‐lineage kinase domain‐like protein (MLKL) is incorporated into the cell membrane, which leads to an influx of extracellular and water molecules resulting in cell swelling and leading to eventual cell lysis.^50^ Researchers have also previously reported that cell rupture occurs during ferroptosis which eventually leads to cellular swelling similarly as in necroptosis.^51^ This is in line with what is observed in our data. Also, the presence of ferroptotic blebs can increase this value.^52^

**TABLE 2:** A summary of area, perimeter, deformability, area ratio, eccentricity, and aspect ratio per condition which were analysed in the L929sAhFas cells.

	Control	Apoptosis (STS 5 h)	Apoptosis (STS 24 h)
Cell area	221 (±40.6)	233 (±78.4)	205 (±92)
Cell perimeter	52 (±5.0)	54 (±10)	50 (±12)
Area ratio	1.030 (±0.017)	1.064 (±0.041)	1.11 (±0.047)
Deformability	0.013 (±0.004)	0.035 (±0.011)	0.031 (±0.014)
Eccentricity	0.52 (±0.081)	0.64 (±0.085)	0.57 (±0.14)
Aspect ratio	1.19 (±0.07)	1.37 (±0.14)	1.24 (±0.16)

For area ratio again a significant increase can be reported for all RCD modalities compared to control cells (*p* < 0.001) indicating that cells undergoing cell death exhibit a rougher morphology. The biggest increases are noticed here for apoptosis and ferroptosis which both have a significantly higher area ratio compared to the necroptotic cells. For apoptotic cells, this is most likely caused by the process of apoptotic blebbing.^49^ Recently, it was reported that a type of macro‐blebbing can also occur during the process of ferroptosis.^52^ In some of the captured images of the ferroptotic cells, these structures are visible (Figure S3). This phenomenon explains the similar increase in the area ratio in ferroptotic cells to apoptotic cells. In the necroptotic cells, a smaller increase is observed. During necroptosis, it is known that the plasma membrane is permeabilized which can lead to irregular shape changes in cells resulting in a higher area ratio.^50^

When looking at the parameters related to the deformation of the cells namely deformability, eccentricity, and aspect ratio, all RCD modalities show a significant increase compared to the control (*p* < 0.001). Care should be taken however since a high area ratio could potentially artificially increase the deformability values (in other research, as a safety an upper limit of 1.05 is set).^41^ For apoptosis, necroptosis and ferroptosis, the area ratio exceeds 1.05. Therefore, to interpret the mechanical changes, the eccentricity has been analysed. The highest increase in eccentricity is noticed in the necroptotic cells, which is significantly higher (*p* < 0.001) than the eccentricity of both apoptotic and ferroptotic cells.

The significant increase in eccentricity compared to the control cells suggests that the cells undergoing RCD are softer and thus deformed more (Figure 2A). A significant increase in eccentricity of necroptotic cells in comparison with apoptotic cells can be noticed, which can be explained by the increase in cellular area of necroptotic cells compared to apoptotic cells. For ferroptosis, a significantly lower eccentricity is measured compared to necroptosis. However, the ferroptotic cells have a significantly higher area compared to the necroptotic cells, thus it would be expected that ferroptotic cells have a higher eccentricity. This can be explained by the aforementioned ferroptotic blebs,^52^ which form irregularities on the cell surface and therefore increase the cellular area but do not contribute to the deformability, since these do not contain intact cytoskeletal protein fibres as was previously shown.^16^

To better understand which parameters are the major contributors to the variability in the data, a principal component analysis was performed (PCA). The first three principal components (PC) explain a cumulative 94% of variance (Table S3). A loadings plot, containing all the data points is presented in Figure 3E (the eigenvalues table is presented in Table S4). In this plot, the first PC is mainly dominated by three parameters namely deformability, eccentricity, and aspect ratio. Furthermore, it can be observed that this principal component analysis allows for data division between RCD induced cells and control cells. The second PC is mainly dominated by area and perimeter. Here no clear division between treatment groups can be observed.

The increase in eccentricity of the cells during the process of cell death is most likely caused by a decrease in elasticity of the cells. This is in line with the AFM measurements (Figure 2A). However, an argument could be made that the area of the RCD induced cells increases and it is this increase of the area that causes the change in deformability, unrelated to the changes in elasticity. To understand the correlation between the different parameters better and find out what parameters are behind the variability in the data a PCA analysis was performed. The first PC, explaining 46% of the variability in the data, is dominated by the three parameters linked to the state of deformation of the cell (deformability, eccentricity, and the aspect ratio). These loadings run very close to each other, indicating a strong correlation between these parameters. While the RCD induced cells score high on PC1, control cells tend to produce negative values on this PC. This indicates the loadings important for this PC1 are good at separating the live from the dead cells. No clear clustering can be observed between different cell death modalities. This also corresponds to the observation in the AFM data, where no clear separation could be made based on the Young's modulus in between different RCD modalities. In addition, the loadings of area and perimeter are very low in PC1 and run almost perpendicular to those of the parameters linked to deformation indicating a very weak to no correlation between these parameters. This observation strongly suggests that the changes in cellular deformation are mainly caused by changes in their elastic properties and to a lesser extent by the changes in morphology. The second PC in this analysis explains 32% of the variance and is mainly dominated by area and perimeter. Contrary to the first PC, here no clear division can be made between RCD induced cells and dead cells. It can however be noticed that the population spread in the control cells is much smaller compared to the RCD induced cells.

### 3.4 sDC for detection of late‐stage RCD

**FIGURE 4:**
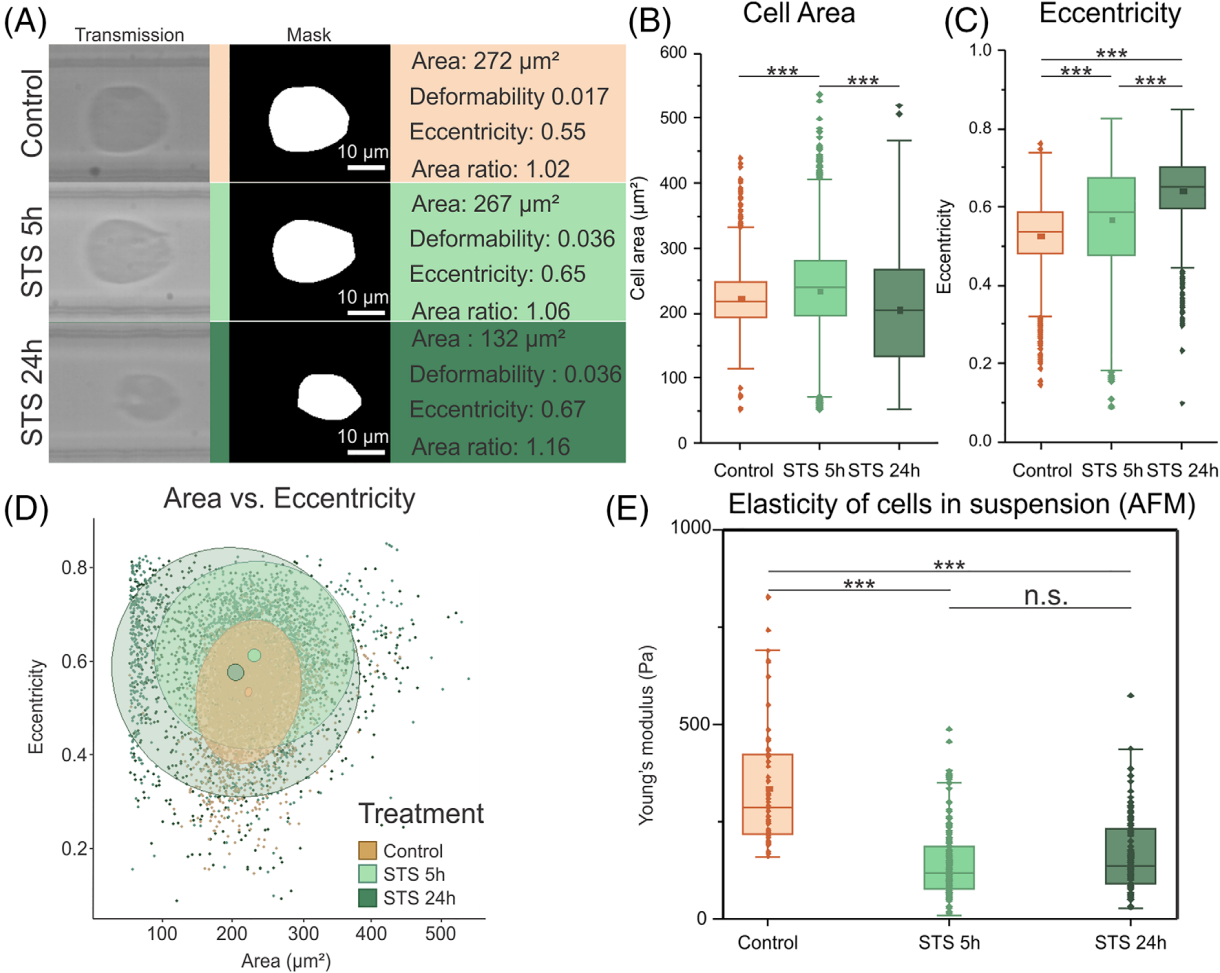
sDC analysis of L929sAhFas cells undergoing apoptosis (STS) in early (5 h) and late stage (24 h). (A) Exemplary images of cell and their corresponding masks from each time point analysed during apoptosis induction, the values indicated next to the cells are directly derived from these masks. (B) Boxplot graph comparing the area of cells after 5 and 24 h of induction of apoptosis (2.5 μM Staurosporin) versus control. (C) Boxplot graph comparing the eccentricity of cells after 5 and 24 h of induction of apoptosis (2.5 μM Staurosporin) versus control. (D) Scatterplot of area in function of eccentricity, the dots indicate the individual cells while the ellipses indicate confidence intervals of the complete data (transparent ellipse represents the 90% confidence interval, solid ellipse represents the 1% confidence interval approximately indicating the mean). (E) Young's modulus values of the cellular elasticities, as measured using AFM, 5 and 24 h after induction of apoptosis using 2.5 μM STS.

Further experiments were performed to verify how this technique performs for later stages of the RCD process. To do this, apoptosis induction in L929sAhFas cells were analysed in the sDC setup at 5 and 24 h. The average values of these parameters are shown in Table 2 (results of the statistical tests are represented in Table S5). Examples of representative cells in these experiments are shown in Figure 4A. When analysing the cell area (Figure 4B) and perimeter, a significant increase can be noticed after 5 h of apoptosis induction (*p* < 0.001). However, at 24 h, a decrease compared to control is observed for both (*p* < 0.001). A potential explanation for this could be the phenomenon of secondary necrosis, which occurs when apoptotic cells are not cleared by immunological cells.^53^ During secondary necrosis the cell membrane is permeabilized and the cellular components start to spill out of the cell. Which eventually leads to an empty hull of a cell membrane left over. Losing all these cellular components also logically leads to decrease in cell area.

At 5 and 24 h after induction, a significant increase in deformability and eccentricity is observed (Figure 4C). When looking at the deformability, it can be noticed that at 5 h after induction and 24 h after induction the values are almost equal, while the eccentricity of the cells at 5 h is significantly higher compared to those at 24 h after induction. As was mentioned previously in this work, for objects with a high area ratio (>1.05) eccentricity should be preferred over deformability^41^ to interpret changes in mechanical properties (this is because of artificial inflation of deformability values caused by surface roughness artefacts). When looking specifically at the area ratio for the different conditions it can be noticed that over time this parameter increases. At 5 h a significant increase from 1.03 (control) to 1.067 can be noted (*p* < 0.001), which further increased at 24 h to an average of 1.11 (*p* < 0.001).

As mentioned above with a high area ratio (>1.05) eccentricity should be preferred over deformability, since eccentricity proves to be a more robust indicator of elasticity for irregularly shaped cells. Figure 4D shows a scatterplot of the cell eccentricity in function of the cell area. After 5 h of induction, the population of cells has shifted upwards towards higher eccentricity values, while maintaining a quasi‐similar area. This suggests that elasticity of cells after 5 h of induction has decreased leading to an increase in eccentricity. There is however still quite some overlap between both the populations. At 24 h after induction, still, a significant increase of eccentricity is present albeit smaller than at 5 h. However, when measuring cells with AFM after 24 h, it is noticed that their elasticity is approximately equal to that of those at 5 h (Figure 4E). The average cell area of cells at 24 h is decreased. Therefore, the cells encounter less shear force if the channel width remains constant. Therefore, it is likely that cells with equal elasticity, but lower cell area will have a decreased eccentricity (which supports our corresponding AFM data; Figure 4E).

It can be noticed on the scatterplot (Figure 4D) that the spread of the population at 24 h is large. Taking into account that we mentioned above that due to secondary necrosis; apoptotic cells lose their contents and only an empty shell remains one should be critical when analysing these results. This deformability cytometry technique was developed for intact cells, and it makes some assumptions in developing this technique namely that the object is an isotropic, linearly elastic sphere, which is not the case anymore in our situation.^40^ From this observation, it can be concluded that, while the deformability cytometry technique might work well for earlier stages in cell death, during the later stages when the cells enter a stage of secondary necrosis, it is likely that the concept of elasticity is lost since one of the essential assumptions of the technique is violated.

## 4 CONCLUSION

In conclusion, the analysis performed here shows that the high‐throughput mechanocytometry technique: sDC can be effectively used to detect mechanical dynamics during the process of several RCD modalities such as apoptosis, necroptosis and ferroptosis. Interestingly, by training SVM on a subset of the sDC experiments, it was possible to make a prediction of alive versus dying state in cells with a high validation accuracy (92%). For apoptosis, necroptosis, and ferroptosis a significant increase in eccentricity could be measured indicating a decrease in elasticity. Furthermore, sDC also allows to detection of other characteristics linked to RCD, such as membrane blebbing, by analysing area ratio. PCA analysis confirmed the hypothesis that the change in eccentricity is indeed caused by changes in mechanical properties. Analysis of apoptosis 24 h after induction also shows promising results for late‐stage RCD analysis. However, caution is necessary since principle assumptions bound to the sDC technique are violated.

Here it was shown that a clear distinction between live and dead cells could be made using sDC, mainly based on the mechanical properties. However, there are some limitations to take into account with this technique. While an indication of the morphology can also be obtained, this is a 2D projection of the 3D real situation. Therefore, other techniques such as SEM, TEM, or AFM are more suited for morphological characterization of cell death modalities.^16^, ^54^, ^55^, ^56^, ^57^ Since it is unsure what the effect of the suspension medium and the induced shear forces are on the cell death process, the samples were not re‐run through the sDC setup. While a clear distinction between viable and dead cells could be made, no further distinction based on mechanical properties was possible in between the different RCD modalities. Further optimization towards recovery of the sample after running could allow to gather dynamics. The dynamics of mechanobiological state of cells enables to distinguish between different RCD modalities which was shown to be possible AFM.^16^ The duration of deformation in the sDC is significantly shorter than that of the AFM (in the range of milliseconds for sDC and seconds for AFM) which could lead to discrepancies in measured (AFM) and the observed deformation (with the sDC).^40^ This is an essential remark since it was mentioned previously that cells are not purely elastic but rather viscoelastic.^58^ Since it has been shown cells behave more like a viscous fluid as they progress further into the process of RCD,^16^ it will take even longer to observe the resulting total deformation, that the shear force of the walls causes on the cells. Knowing this it is possible that the real difference in deformability between control cells and dead cells is still larger than measured in the results. Thus, essential information on the viscoelastic state of cell in this technique is missing. However, new avenues are being explored that would enable this in the future.^59^

Recently, advancements have led to development of a sorting system linked to deformability cytometer with the possibility of measuring fluorescence signal, enabling a user to sort samples based on their mechanical properties and fluorescent labelling(with a throughput up to 200 cells/second).^39^ Combining this sorting technique with the data presented in this work provides strong outlook for future applications. Dead cells tend to have a higher unspecific uptake of probes, altered antigen expression^60^ or increased autofluorescence.^61^ The ability to remove dead cells from a sample in a label‐free manner (based on their mechanical properties) before staining can greatly increase the quality of the results. Inversely, for other applications, the early‐stage dead cells are of significant interest. An example of this can be found in testing the immunogenicity of early‐stage ferroptotic cells.^57^ The data shown in this work show the perspective to split such a sample into two categories: early‐stage dead cells and viable cells allowing to exclude more variability in the data. Since these systems work with immune responses, any foreign components (such as fluorescent labels) should be avoided, thus showing the need for effective label‐free techniques.

## AUTHOR CONTRIBUTIONS

Louis van der Meeren, André G. Skirtach, Joost Verduijn: Conceptualization. Louis van der Meeren, André G. Skirtach: Methodology. Louis van der Meeren, Joost Verduijn: Investigation. Louis van der Meeren: Visualization. André G. Skirtach, Dmitri V. Krysko: Supervision. Louis van der Meeren: Writing – original draft. Louis van der Meeren, Joost Verduijn, André G. Skirtach, Dmitri V. Krysko: Writing – review and editing.

## FUNDING INFORMATION

This work was developed under strategic basic research funding FWO‐SBO (S001423N) and the Ghent University grants (BOF01/ O3618, BOF/IOP/2022/033, BOF23/GOA/029). We also acknowledge the support of FWO‐Flanders (I002600N) and FWO‐F.N.R.S under the Excellence of Science (EOS) program, grant number 40007488.

## CONFLICT OF INTEREST STATEMENT

The authors declare no competing interests.

## Supporting information


**Data S1:** Supporting Information

## Data Availability

All data and scripts used are available on the online archive Mendeley data, DOI: 10.17632/328zjkn4fh.1.
