# Supplementary material for: High‐throughput mechano‐cytometry as a method to detect apoptosis, necroptosis, and ferroptosis
Source: Cell Prolif. 2023 Mar 29;56(6):e13445. doi: 10.1111/cpr.13445 (PMC10280138; doi:10.1111/cpr.13445)
Supplement: Supplementary file 1 — Data S1: Supporting Information [file CPR-56-e13445-s001.docx]

***Supplementary materials***

***Supplementary Figure 1: Cell death kinetics experiment***

***Supplementary Figure 2: Detached cell size determination***

***Supplementary Figure 3: Ferroptotic blebs***

***Supplementary Table 1: sDC parameters formulations***

***Supplementary Table 2. Table containing P-values from the statistical analysis of apoptosis, necroptosis and ferroptosis induced in L929sAhFas:***

***Supplementary Table 3. Summary table PCA variances:***

***Supplementary Table 4: P-values from the statistical analysis of ferroptosis induced in MCA205 cells***

*
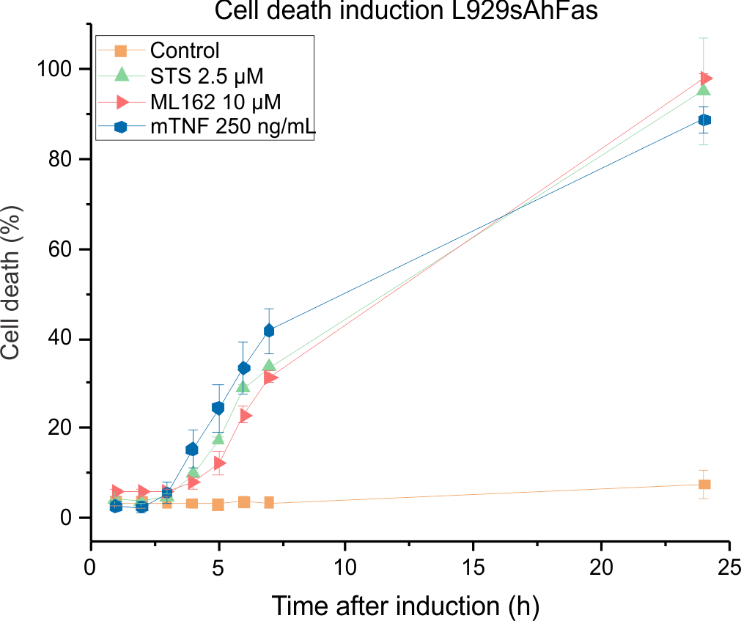
*

**Supplementary Figure 1 Cell death kinetics experiment** Line graph showing an over-time cell death induction experiment. Cell death was analysed at multiple time points after addition of different cell death inducers. Percentage of dead cells was analysed by using Propidium iodide, indicating end stage cell death when membrane permeabilization has occurred.

**
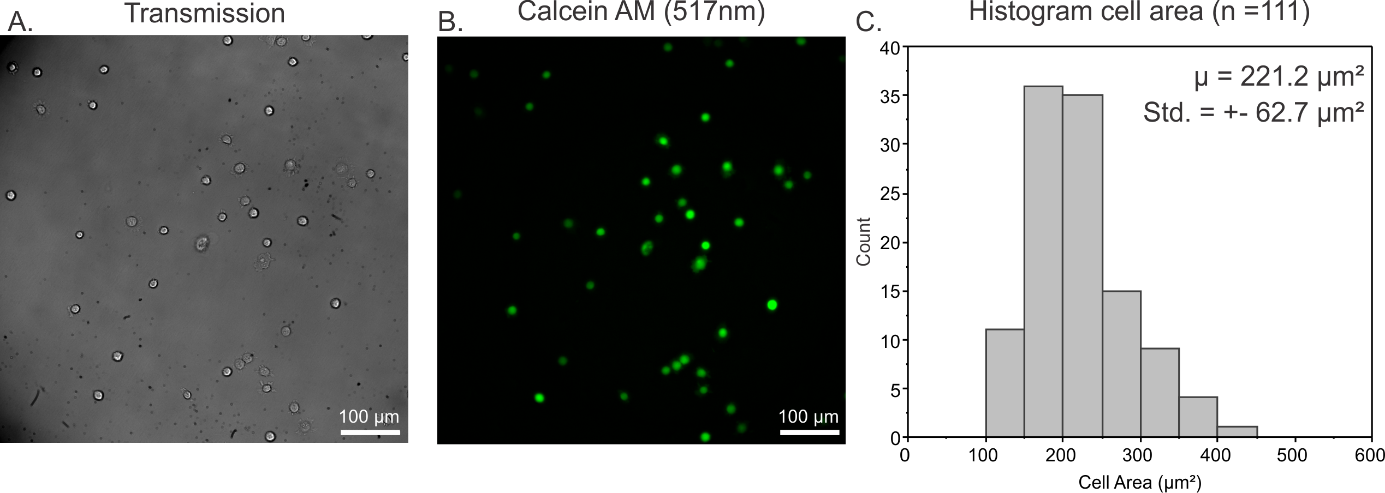
Supplementary Figure 2 Detached cell size determination A.** Transmission and **B.** Fluorescence microscopy image used to determine the average area of a L929sA-hFas cells detached from the surface. Calcein AM is used as a fluorescent label to label the whole cell, allowing to determine cell area. **C.** Using this data from multiple images, an average cell area of control cells could be determined shown on the histogram


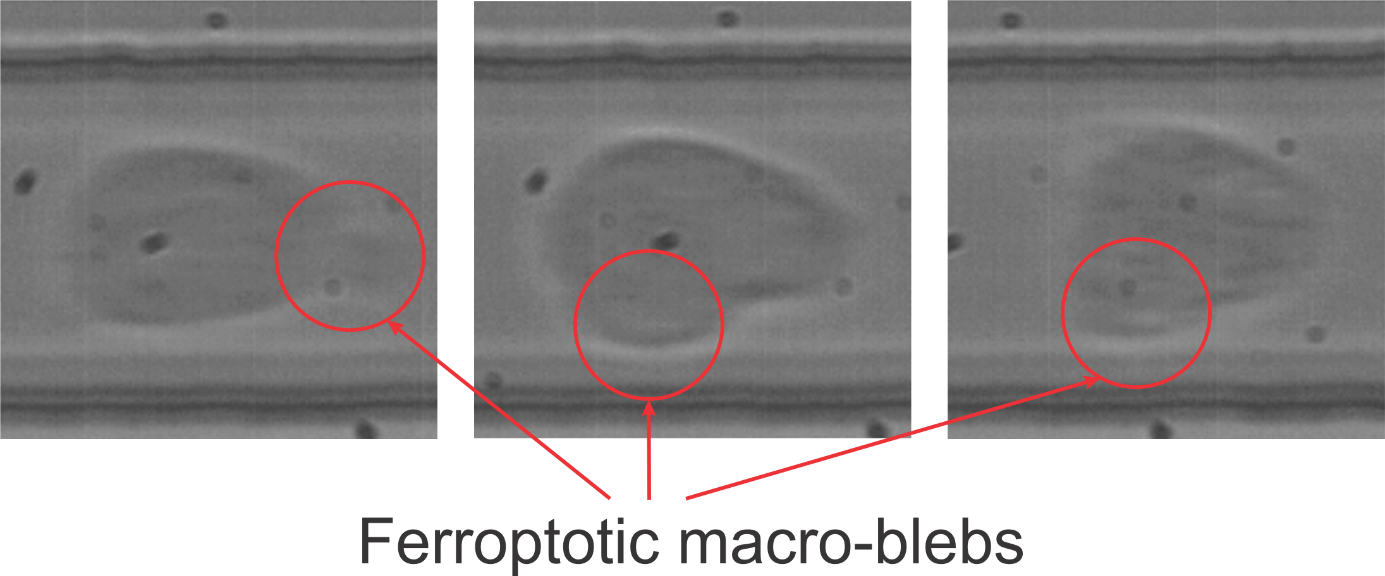


***Supplementary Figure 3 Ferroptotic blebs:*** *Images indicating blebs present in ferroptotic cells induced for 5 hours using ML162.*

***Supplementary table 1. sDC parameters: Explanation and formulae for the parameters that are extracted from the transmission images and used for data interpretation.***

| **Parameter** | **Explanation** | **Formula** |
| --- | --- | --- |
| **Area** | Actual cell area which is detected by the cell mask. This value is obtained by taking the sum of all pixels in the mask and multiplying it with the image scale. |  |
| **Perimeter** | Perimeter of the cell area. This value is obtained by calculating the amount of pixels at the edge of the cell mask and multiplying this with the image scale |  |
| **Convex hull** | Smallest convex polygon that can contain the actual cell area. |  |
| **Area ratio** | Value obtained by taking the ratio of the convex hull area over the actual measured cell area. This parameter can be used as a parameter of object roughness/irregularity. As the object is smoother, this value will evolve to 1 and vice versa | $R=\frac{A_{hull}}{A_{contour}}$ |
| **Circularity** | Parameter indicating the roundness of an object. A perfect circle has the value 1. | $C=\left( \frac{2*\sqrt{\left( \pi A \right)}}{P} \right)$ |
| **Deformability** | Parameter indicating the deviation of roundness of an object. This value is defined as 1-circularity | $D=1-C=1-\left( \frac{2*\sqrt{\left( \pi A \right)}}{P} \right)$ |
| **Eccentricity** | Eccentricity of the ellipse that has the same second-moments as the region, returned as a scalar. The eccentricity is the ratio of the distance between the foci of the ellipse and its major axis length. The value is between 0 and 1. (0 and 1 are degenerate cases. An ellipse whose eccentricity is 0 is actually a circle, while an ellipse whose eccentricity is 1 is a line segment.) | $Ecc=\frac{\sqrt{a^{2}-b^{2}}}{a}$  $a=major axis length$  $b=minor axis length$ |
| **Normalized average grey value** | Mean of all the grey values in the masked cell area, returned as a scalar. This value is normalized using the background to buffer for brightness differences in between different experiments. | $Avg_{grey}=\frac{mean\left( grey_{cell} \right)-mean\left( grey_{background} \right)}{Std\left( grey_{background} \right)}$ |
| **Coefficient of Variation of gray value** | Standard deviation of the grey values in the masked cell area divided by the mean of the grey values in the masked cell area. | $C.V._{grey}=\frac{\left( Standard deviation\left( grey_{cell} \right) \right)}{mean\left( grey_{cell} \right)}$ |
| **Aspect ratio** | Ratio of the x-length over the y-length of the bounding box that can be drawn around the masked cell area |  |

***Supplementary Table 2. Table containing P-values from the statistical analysis of apoptosis, necroptosis and ferroptosis induced in L929sAhFas:*** *The first column represents the parameter that is analysed, the first row indicates which conditions are compared. All tests are performed using a pairwise t-test using Bonferroni adjustment for the p-values to correct for multiple testing. Values in bold indicate significant differences.*

|  | **Area** | **Perimeter** | **Deformability** | **Area ratio** | **Eccentricity** | **Aspect Ratio** |
| --- | --- | --- | --- | --- | --- | --- |
| **Control-ML162** | **<2e-16** | **<2e-16** | **<2e-16** | **<2e-16** | **<2e-16** | **<2e-16** |
| **STS-Control** | **7.4e-10** | **5.3e-15** | **<2e-16** | **<2e-16** | **<2e-16** | **<2e-16** |
| **mTNF-Control** | **<2e-16** | **<2e-16** | **<2e-16** | **<2e-16** | **4.55E-03** | **<2e-16** |
| **ML162-mTNF** | ***2.3e-08*** | ***2.0e-08*** | **<2e-16** | 0.13 | 0.31 | 0.05489 |
| **STS-mTNF** | **1.0e-13** | **2.7e-15** | **4.5e-11** | **<2e-16** | **<2e-16** | **0.00098** |
| **ML162-STS** | **<2e-16** | **<2e-16** | **2.4e-05** | **<2e-16** | **1.5e-13** | **5.8e-11** |

***Supplementary Table 3. Summary table PCA:*** *This table contains the summary of the PCA performed on the sDC data. For each principle component the standard deviation, proportion of variance and cumulative proportion of variance is presented.*

| **Importance of components:** | **PC1** | **PC2** | **PC3** | **PC4** | **PC5** | **PC6** |
| --- | --- | --- | --- | --- | --- | --- |
| **Std** | *1.6702* | *1.3858* | *0.9898* | *0.42999* | *0.34019* | *0.0975* |
| **Proportion of variance** | *0.4649* | *0.3201* | *0.1633* | *0.03082* | *0.01929* | *0.00158* |
| **Cumulative proportion** | *0.4649* | *0.785* | *0.9483* | *0.97913* | *0.99842* | *1* |

***Supplementary Table 4. Eigenvalues table PCA:*** *Here the values of the individual loadings for each of the principle components are presented.*

|  | **Area** | **Perimeter** | **Area ratio** | **Deformability** | **Eccentricity** | **Aspect ratio** |
| --- | --- | --- | --- | --- | --- | --- |
| **PC1** | *0.25* | *0.64* | *-0.17* | *0.03* | *0.01* | *0.70* |
| **PC2** | *0.27* | *0.63* | *-0.16* | *-0.01* | *-0.02* | *-0.71* |
| **PC3** | *0.52* | *-0.24* | *-0.16* | *-0.71* | *0.37* | *0.03* |
| **PC4** | *0.16* | *-0.29* | *-0.87* | *0.34* | *-0.14* | *-0.01* |
| **PC5** | *0.53* | *-0.14* | *0.31* | *0.62* | *0.48* | *-0.02* |
| **PC6** | *0.54* | *-0.16* | *0.27* | *-0.02* | *-0.78* | *0.03* |

**Supplementary Table 5.** Table containing P-values from the statistical analysis of early and late-stage apoptosis (5 hours and 24 hours) induced in L929sAhFas: The first column represents the parameter that is analyzed, the first row indicates which conditions are compared. All tests are performed using a pairwise t-test using Bonferroni adjustment for the p-values to correct for multiple testing. Values in bold indicate significant differences.

|  | Area | Perimeter | Deformability | Area ratio | Eccentricity | Aspect Ratio |
| --- | --- | --- | --- | --- | --- | --- |
| Control-STS_24h | **6.00E-14** | **2.00E-16** | **<2e-16** | **1.00E-07** | **<2e-16** | **4.90E-16** |
| Control-STS_5h | **3.50E-09** | **1.10E-12** | **<2e-16** | **1.00E-07** | **<2e-16** | **<2e-16** |
| STS_5h-STS_24h | **<2e-16** | **<2e-16** | **<2e-16** | **<2e-16** | **<2e-16** | **<2e-16** |
